# Zhongxian Lin: Founder of color psychology in China

**DOI:** 10.1007/s13238-017-0420-8

**Published:** 2017-05-12

**Authors:** Hai-Feng Li, Xiao-Mei Li, Bu-Xin Han

**Affiliations:** 1 Department of Psychology, Fujian Normal University, Fuzhou, 350117 China; 2 Key Lab of Mental Health, Institute of Psychology, Chinese Academy of Sciences, Beijing, 100101 China

**Figure 1:**
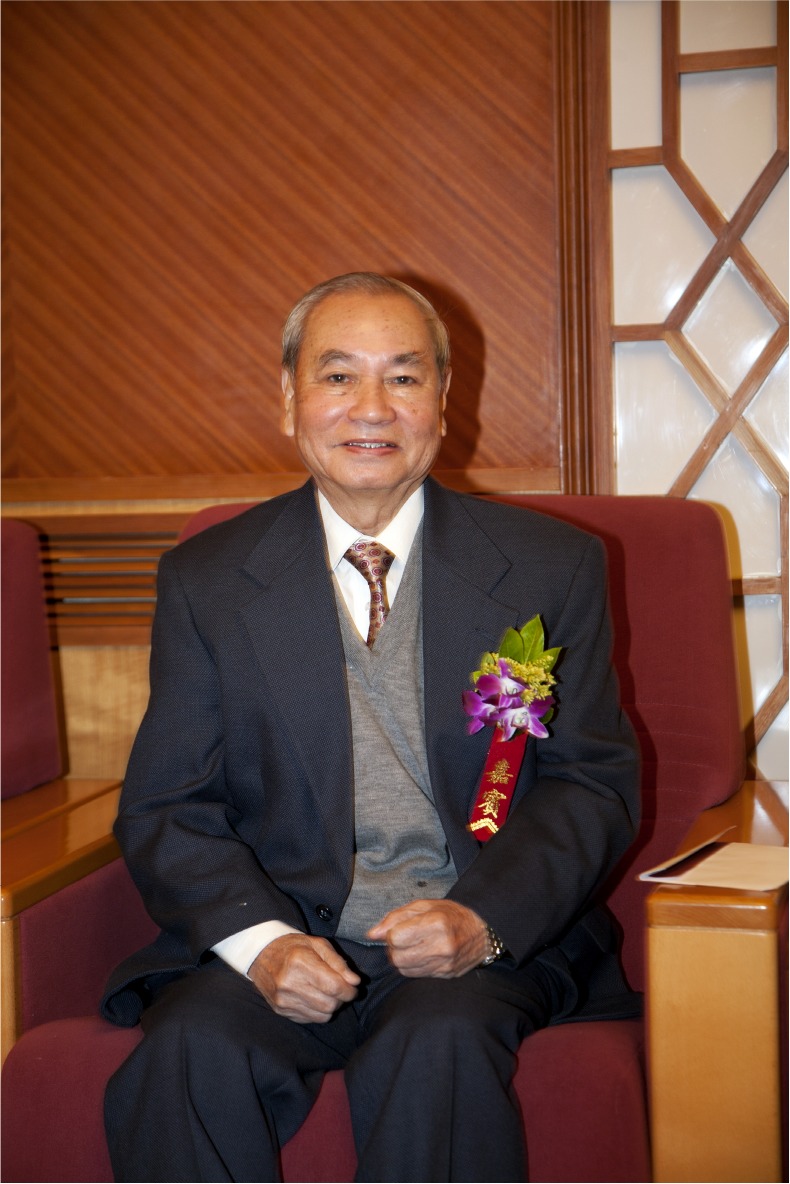
Professor Zhongxian Lin (1931–2011) (Fu, 2012)

 Professor Zhongxian Lin (林仲贤, 1931–2011) is the founder and the leader of color psychology in China. He made pioneering researches on the determination of the color vision and the Chinese skin color. He is a member of executive committee of the AFRO-ASIAN Psychological Association (AAPA), the National Color Standardization Committee, the New York Academy of Sciences, and the American Association for the Advancement of Science (AAAS) (Fig. 1).

Born on December 3rd, 1931 in Guangzhou, Professor Lin spent his childhood there. After the outbreak of Sino-Japan war in 1937, he took refuge with his mother in their hometown, Enping county. After the victory of the War in 1945, he came back and finished his senior high school in Guangzhou. In 1951, he went to Tsinghua University to study psychology, and continued his study in Peking University the next year. During that time, he took a broad range of courses, including psychology, philosophy and physiology, which laid a solid foundation for his future researches. In 1955, he graduated from Peking University with honors, and was assigned to work at the Institute of Psychology, Chinese Academy of Sciences (IPCAS). There, he was promoted as professor in 1986.

At the beginning of his research career at IPCAS, Professor Lin studied aviation psychology, which was supervised by Professor Richang Cao (曹日昌). He published more than 10 articles in several journals in this area, such as *Acta Psychologica Sinica* and *Aviation Knowledge*. Meanwhile, he participated in the key research projects collaborated with military forces, and published 3 review articles. He elaborated the mechanism of altitude estimation during aircraft landing (Lin, 1961), and stated that one of the main reasons for aircraft accidents was attributed to the difficulty for the pilot to estimate the altitude of the aircraft when landing. He proposed that estimation of landing distance is a complex conditioned reflex behavior, and a unified comprehensive dynamic stereotype. Professors Zhongxian Lin and Qicheng Jing (荆其诚) systematically summarized vision problems under different flight conditions, such as high speed, low speed, oversea and night flights, as well as the eye movement problems during the flights (Lin & Jing, 1962). In addition, he suggested that methods of experimental psychology should be utilized to predict the cognitive activities and motor responses when selecting pilots and case analysis should be used to evaluate the candidates’ emotion, will, and personality (Jing & Lin, 1962). Working together with Professors Richang Cao and Genquan Feng (封根泉), Professor Lin’s research on *The experimental study of preventing and overcoming flight illusion* (1965) won the Prize of the Science and Technology Achievement Award of the Ministry of Defense, and the Significant Achievement Prize of the Chinese Academy of Sciences.

**Figure 2:**
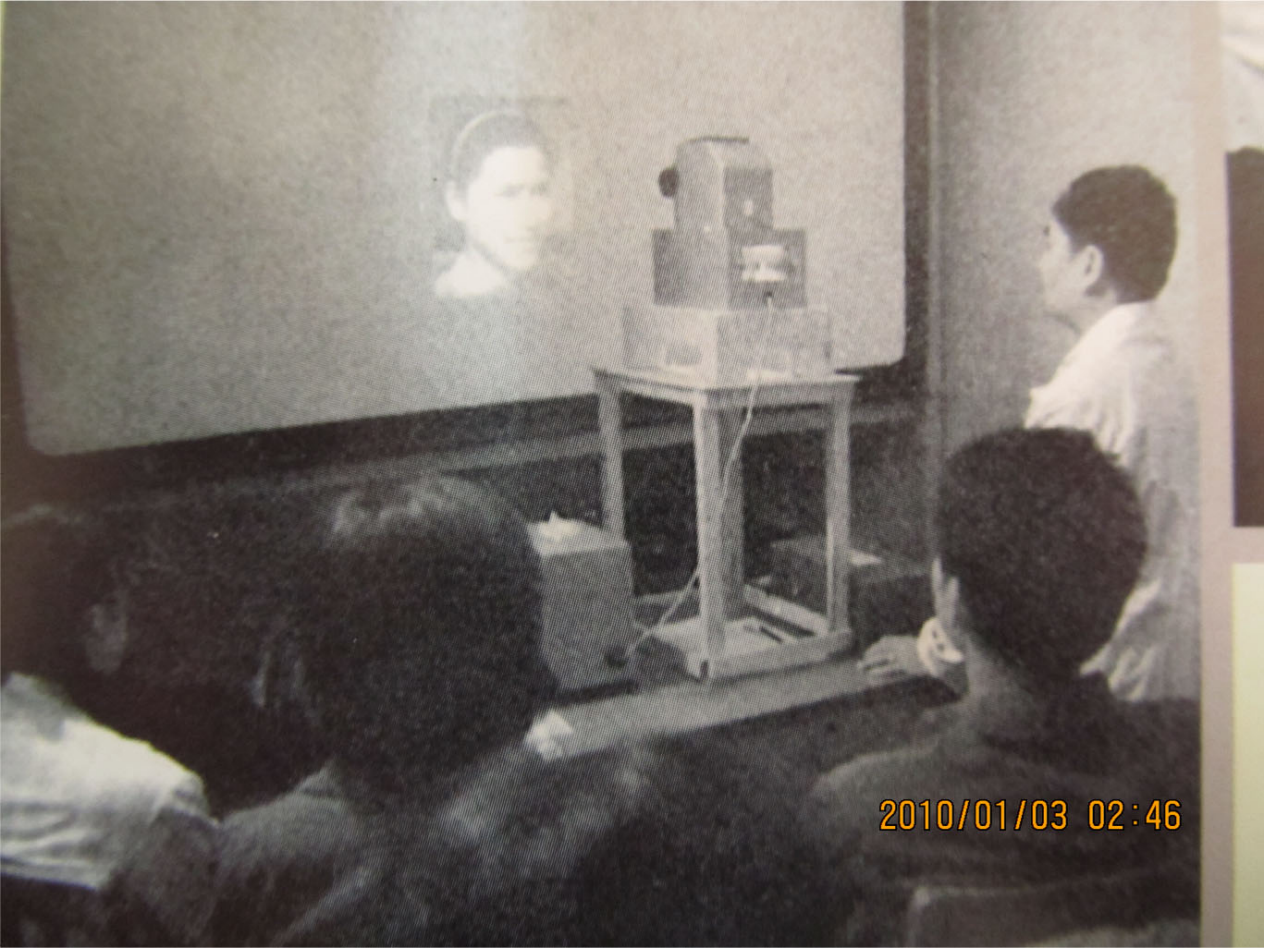
Skin color measurement (Fu, 2012)

On the initiative of Professor Lin and his colleagues, IPCAS was reestablished in 1972 after the “Cultural Revolution”. At that time, Professor Lin started his research on color psychology which studies the production, acceptance and application of color, and its standardization (Fig. 2). He published a series of representative books, such as *Vision and Color Measurement Application*, and *Psychology of Color Vision*, and representative articles, such as “Chinese skin color measurement”, and “Color naming and perceptual discrimination of Chinese preschool children”. He hosted and participated in the determination of several national standards, including *Reflection Colour Bar Test Card* (1987), *Reflection Flesh Tone Test Card* (1987), *Transparency Colour Bar Test Card* (1987), *Transparency Flesh Tone Test Card* (1987), *Colour TV Receiver-Colour Temperature of White Point-Reference White and Its Tolerance* (1989), *Colors of Light Signals* (1987), *Method for Visual Evaluation of Metamerism* (1995) and *The Chinese Color System* (1995). These pioneering achievements provided significant theoretical basis and national standards for the development of several professional areas, such as psychology, photology, film, and television. For his outstanding achievements on color psychology, after Chairman Mao’s death in September, 1976, Professor Lin was invited to measure Chairman Mao’s skin color for the body reservation.

Besides aviation psychology and color psychology, Professor Lin also opened up a new avenue for the psychological researches on working on the plateau. He proved that drivers’ sensory and brain functions can be affected by the low-oxygen at the altitude around 4,000 meters (Lin et al., 1981). He also found that depth perception error increased with the increase of altitude (Lin, 1986). Whether in the plains or on the plateau, high work load would result in decreased depth perception function of the drivers. He also summarized and analyzed the physical and mental status of the human beings on the plateau, including the influences of low-oxygen on sensory perception, complex mental activity, and central nervous system (Lin, 1988). These studies made substantial contributions to the development of scientific research in the plateau areas.

As a leading scientist for the researches on the Chinese psychology, Professor Lin devoted almost all his life to the recovery and development of the Chinese Psychological Society (CPS). He served as the deputy secretary general (1984–1989) and the secretary general (1989–1993) for the CPS, and was elected as the sixth President of the CPS in October, 1993 based on his outstanding contributions to the field and his excellent capabilities on leadership, organization, and coordination. He investigated the developmental status of psychology in the majority of provinces in China, and made the significant layout for the development of Chinese psychology, which drove the development of the CPS considerably.

In addition, Professor Lin actively promoted the Chinese psychology to the world. He attended the International Congress of Psychology several times, and carried out academic exchanges with more than 10 countries, and introduced the current situations and achievements of the Chinese psychology to the world. Due to his prominent contributions, he was awarded the Lifetime Achievement Award of the CPS, and was elected as the Fellow of the CPS in 2004. He was included in *The Scientists directory of the Chinese Academy of Sciences*, *Who*’*s Who of the Asian Pacific Rim*, *The Brief Biographies of Chinese Contemporary Education Celebrities*, *Dictionary of Contemporary Chinese Scientists and Inventors*, *Who*’*s Who of the International Biographical Centre of Cambridge*, *Who*’*s Who of the American Biographical Centre*. His outstanding contributions have been recorded in the history of the Chinese psychology.
